# Pyruvate kinase M2 regulates photoreceptor structure, function, and viability

**DOI:** 10.1038/s41419-018-0296-4

**Published:** 2018-02-14

**Authors:** Ammaji Rajala, Yuhong Wang, Richard S. Brush, Kristine Tsantilas, Connor S. R. Jankowski, Ken J. Lindsay, Jonathan D. Linton, James B. Hurley, Robert E. Anderson, Raju V. S. Rajala

**Affiliations:** 1 Department of Ophthalmology, University of Oklahoma Health Sciences Center, Oklahoma City, OK USA; 2 Dean McGee Eye Institute, Oklahoma City, OK USA; 3 Department of Biochemistry and Ophthalmology, University of Washington, Seattle, WA USA; 4 Department of Cell Biology, University of Oklahoma Health Sciences Center, Oklahoma City, OK USA; 5 Department of Physiology, University of Oklahoma Health Sciences Center, Oklahoma City, OK USA

## Abstract

Pyruvate kinase M2 (PKM2) is a glycolytic enzyme that is expressed in cancer cells. Its role in tumor metabolism is not definitively established, but investigators have suggested that regulation of PKM2 activity can cause accumulation of glycolytic intermediates and increase flux through the pentose phosphate pathway. Recent evidence suggests that PKM2 also may have non-metabolic functions, including as a transcriptional co-activator in gene regulation. We reported previously that PKM2 is abundant in photoreceptor cells in mouse retinas. In the present study, we conditionally deleted PKM2 (rod-cre PKM2-KO) in rod photoreceptors and found that the absence of PKM2 causes increased expression of PKM1 in rods. Analysis of metabolic flux from U-^13^C glucose shows that rod-cre PKM2-KO retinas accumulate glycolytic intermediates, consistent with an overall reduction in the amount of pyruvate kinase activity. Rod-cre PKM2-KO mice also have an increased NADPH availability could favor lipid synthesis, but we found no difference in phospholipid synthesis between rod-cre PKM2 KO and PKM2-positive controls. As rod-cre PKM2-KO mice aged, we observed a significant loss of rod function, reduced thickness of the photoreceptor outer segment layer, and reduced expression of photoreceptor proteins, including PDE6β. The rod-cre PKM2-KO retinas showed greater TUNEL staining than wild-type retinas, indicating a slow retinal degeneration. In vitro analysis showed that PKM2 can regulate transcriptional activity from the PDE6β promoter in vitro. Our findings indicate that both the metabolic and transcriptional regulatory functions of PKM2 may contribute to photoreceptor structure, function, and viability.

## Introduction

Unlike normal cells, which use glucose for the synthesis of ATP through oxidative phosphorylation, cancer cells utilize glucose to synthesize macromolecular building blocks, such as lipids, proteins, and nucleic acids^1,2^. This fuel redirection is thought to be mediated by pyruvate kinase M2 (PKM2), a glycolytic enzyme that dephosphorylates phosphoenolpyruvate (PEP) to pyruvate, the last step in glycolysis^3^. In yeast cells, reduced PKM2 activity has been shown to activate the pentose phosphate pathway (PPP) through accumulation of PEP^4^. This relationship has also been demonstrated in tumor cells; PKM2 activity that has been reduced by tyrosine-105 phosphorylation increases glycolytic intermediates and NADPH generation, favoring the diversion of glucose flux towards macromolecular synthesis^5^.

We previously reported that PKM2 is the major isoform expressed in rod photoreceptor cells^6^. Photoreceptor cells are postmitotic and highly metabolic; their energy expenditure is comparable to that of multiplying tumor cells^1,2,7,8^. On a daily basis, photoreceptors shed 10% of their outer segment (OS) distal tips to maintain their length for proper function. As a result, these cells must synthesize nucleic acids for mRNA production, and lipids and proteins for the daily renewal of photoreceptor membranes^9^. We and others hypothesized that high PKM2 expression in photoreceptors redirects glucose to anabolic processes and the activation of the PPP needed for NADPH generation. NADPH is essential for membrane synthesis, antioxidant metabolism (reduction of oxidized glutathione), and reduction of all-*trans*-retinal formed from the photoisomerization of 11-*cis*-retinal as a result of rhodopsin activation^6,10^. In photoreceptors, PKM2 undergoes a light-dependent Y105 phosphorylation^6^, but the significance of this phosphorylation in photoreceptor functions is currently unknown. Moreover, the functional role of PKM2 in photoreceptor cells is unclear. Recent evidence from studies of cancer cells suggests that PKM2 can perform non-canonical or non-metabolic functions as a transcriptional co-activator in gene regulation^11,12^. As PKM2 is predominantly expressed in the photoreceptor cells of both rods and cones^6^, we examined the effect of PKM2 loss on rod photoreceptor structure, function, and viability.

## Results

### Conditional deletion of PKM2 in rod photoreceptor cells

**Fig. 1:**
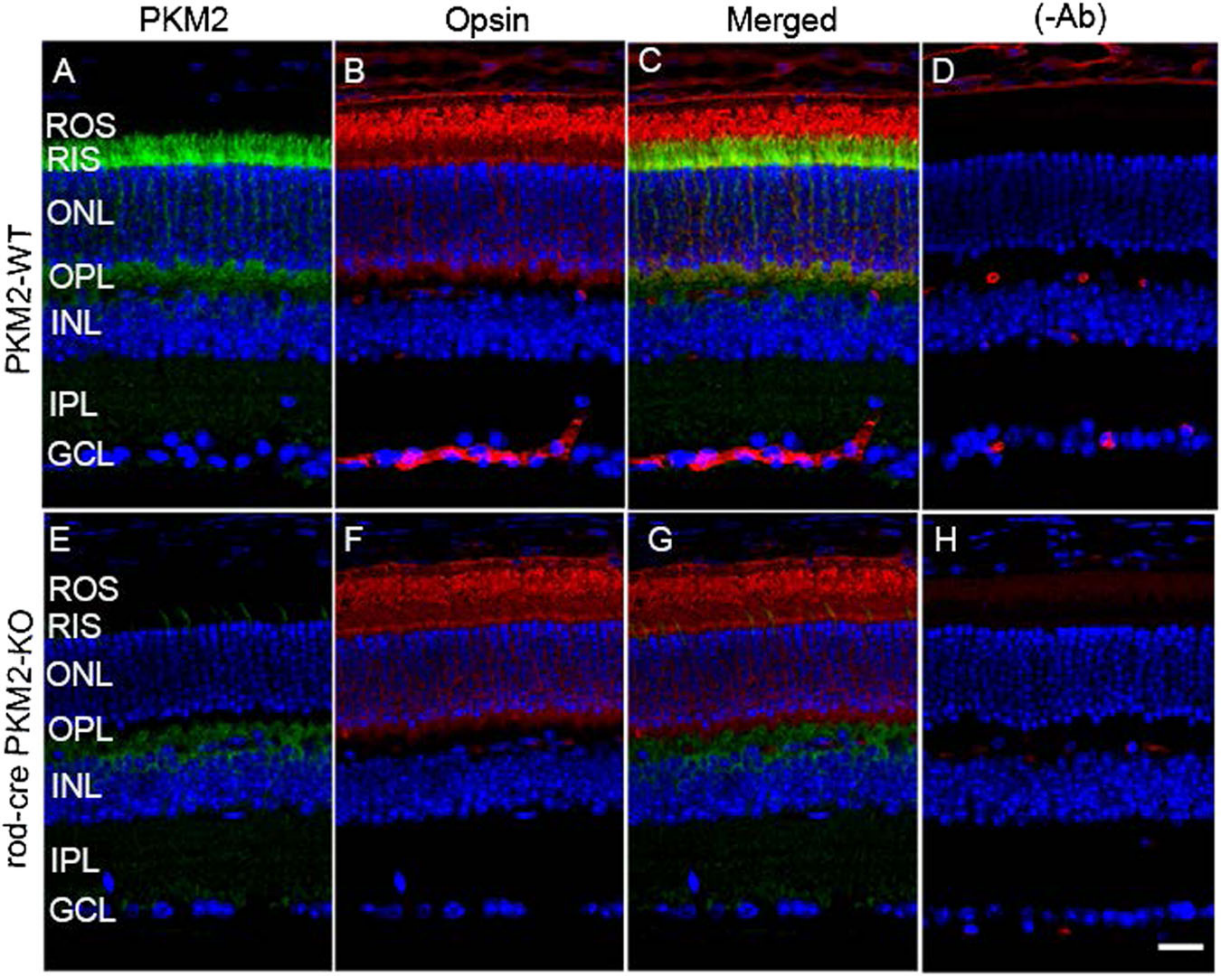
Immunofluorescence analysis of PKM2 in wild-type and rod-cre PKM2-KO mice. Prefer-fixed sections of wild-type **a**-** d** and rod-cre PKM2-KO **e**–**h** mouse retinas were subjected to immunofluorescence with anti-PKM2 **a**,** e** and anti-opsin **b**,** f**. **c**,** g** Merged images of PKM2 and opsin. **d**, **h** Omission of primary antibodies. GCL, ganglion cell layer; INL, inner nuclear layer; IPL, inner plexiform layer; ONL, outer nuclear layer; OPL, outer plexiform layer; RIS, rod inner segment; ROS, rod outer segments. Scale bar = 50 μm

**Fig. 2:**
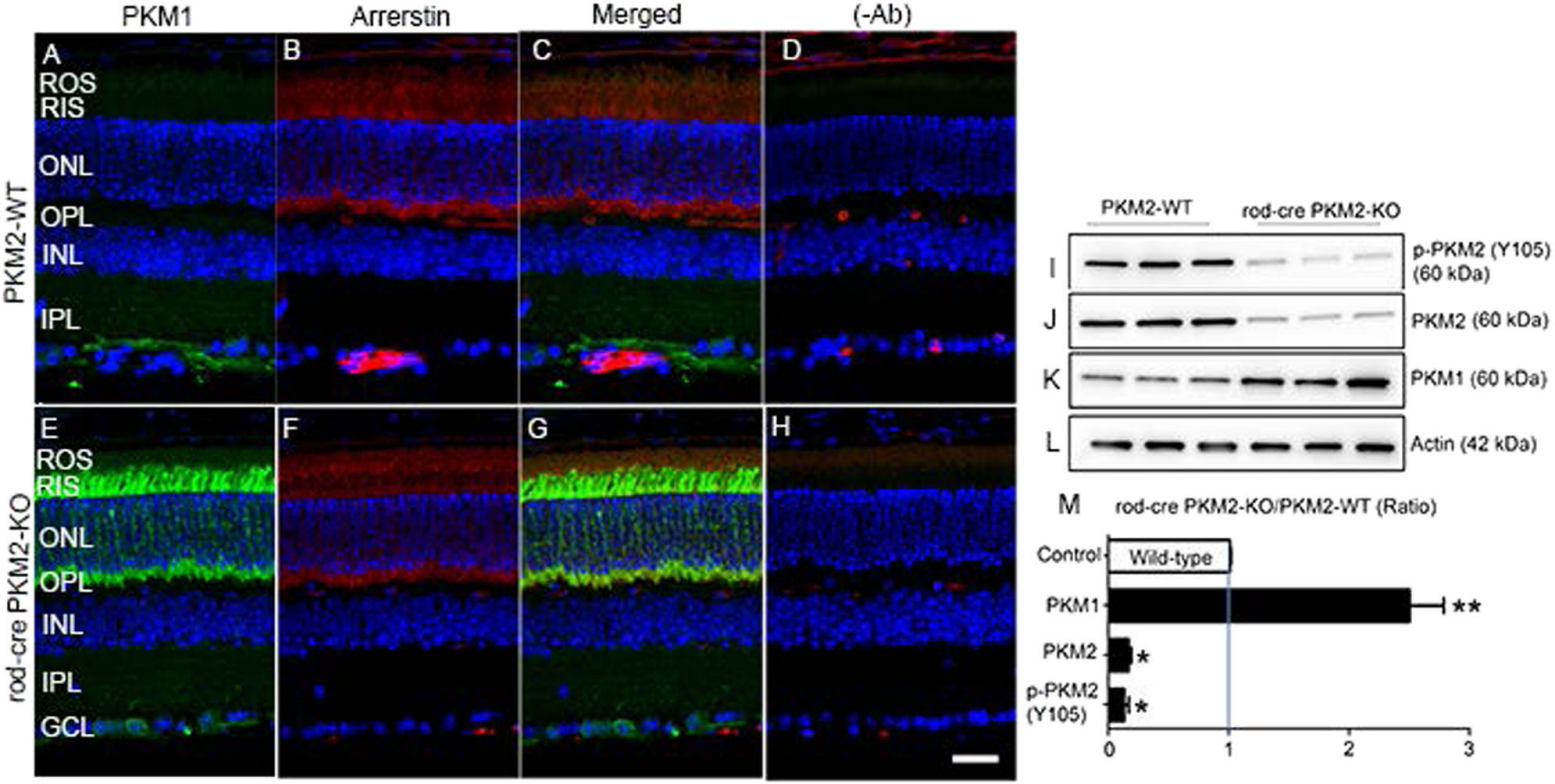
Immunofluorescence analysis of PKM1 in wild-type and rod-cre PKM2-KO mice. Prefer-fixed sections of wild-type **a**-** d** and rod-cre PKM2-KO **e**–**h**) mouse retinas were subjected to immunofluorescence with anti-PKM1 **a**,** e** and anti-arrestin **b**,** f** antibodies. **c**, **g** Merged images of PKM1 and arrestin. **d**, **h** Omission of primary antibodies. GCL, ganglion cell layer; INL, inner nuclear layer; IPL, inner plexiform layer; ONL, outer nuclear layer; OPL, outer plexiform layer; RIS, rod inner segment; ROS, rod outer segments. Scale bar = 50 μm. Retinal homogenates (5.0 µg protein) from wild-type and rod-cre PKM2 KO mice were subjected to immunoblot analysis with anti-pPKM2 (Y105) **i**, anti-PKM2 **j**, anti-PKM1 **k**, and anti-actin **l** antibodies. We normalized the protein expression/phosphorylation to actin **m** and then calculated the ratios (rod-cre PKM2-KO/PKM2-WT). Data are mean ± SEM (*n = *3). *p < 0.004, **p < 0.001

To determine the functional role of PKM2 in rod photoreceptor cells, we mated the exon 10 floxed PKM2 mice with a transgenic mouse line expressing Cre-recombinase under the control of a 4 kb mouse rod opsin promoter (i75-Cre)^13^. Retinal sections from 2-month-old PKM2 wild-type and rod-cre PKM2-KO mice were immunostained with anti-PKM2 and anti-opsin antibodies. The results revealed the expression of PKM2 in the inner segments and outer plexiform layer of photoreceptor cells in wild-type mice (Fig. 1A,C). In the rod-cre PKM2-KO mice, there was no expression of PKM2 in the inner segments (Fig. 1E). We found that opsin properly localized to rod OSs (ROSs) in both PKM2 wild-type (Fig. 1B,C) and rod-cre PKM2-KO mice (Fig. 1F,G), suggesting that there was no indication of rod photoreceptor degeneration or mislocalization of opsin in mice at 2 months of age. It has been shown previously that splicing repressors suppresses the inclusion of exon 9 (PKM1) and favors inclusion of exon 10 (PKM2)^14^. In PKM2 floxed mice, the exon 10 was floxed and cre-mediated excision resulted in the complete loss of PKM2, which resulted in the upregulation of PKM1 in photoreceptor cells (Fig. 2E,G) compared with PKM2-WT mouse retina (Fig. 2A,C). This experiment suggests that loss of PKM2 upregulates PKM1 in rod photoreceptor cells. Immunoblot analysis show a significant loss of PKM2 protein and PKM2 phosphorylation in rod-cre PKM2-KO mice compared with wild-type mice (Fig. 2I,J,M). Consistent with the results of immunohistochemistry, the PKM1 expression was significantly higher ( 2.5-fold) in the rod-cre PKM2-KO mice than in the wild-type mice (Fig. 2K,M).

**Fig. 3:**
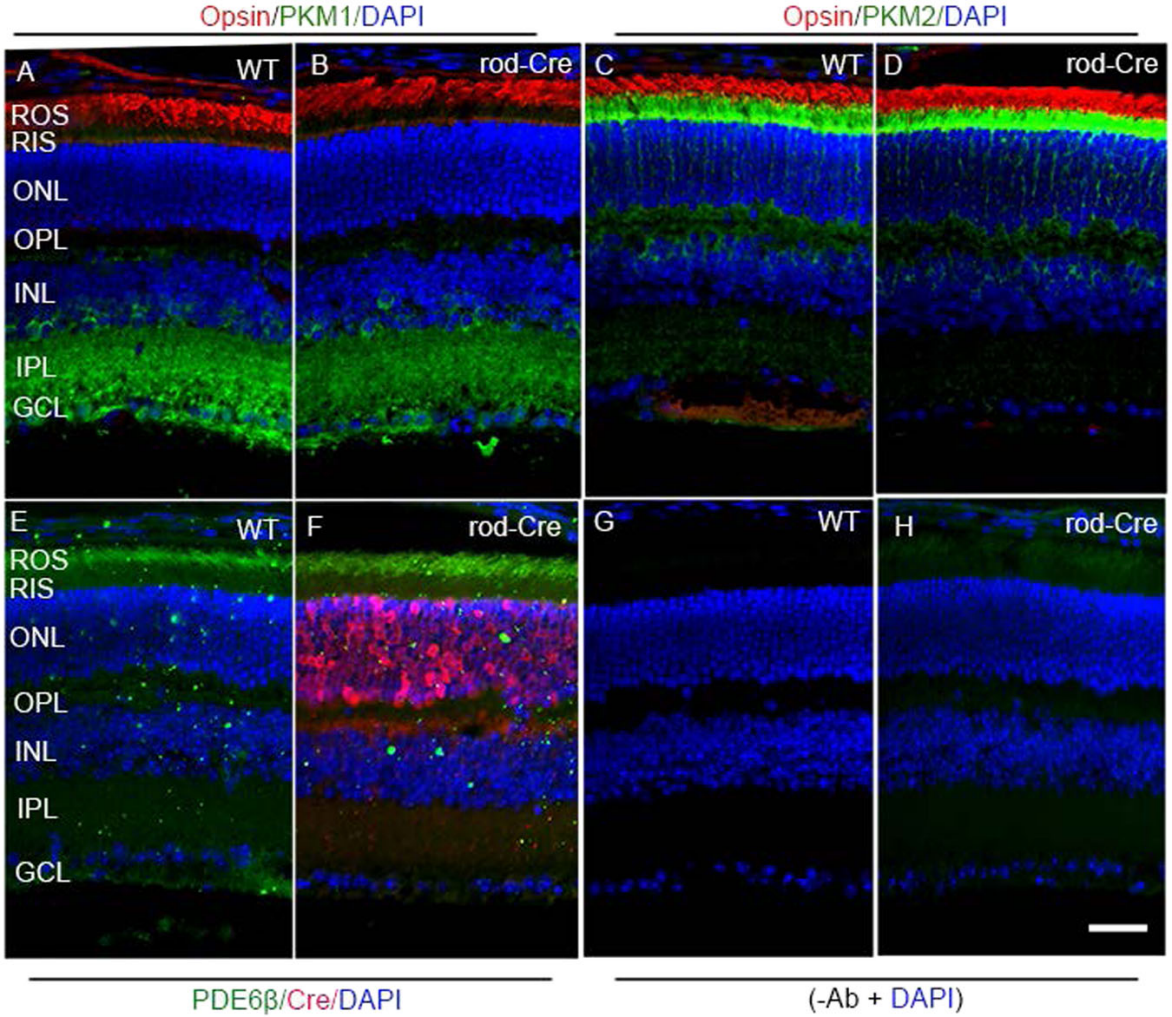
Expression of PKM1, PKM2, and rod photoreceptor marker proteins opsin and PDE6β in wild-type and rod-cre mice. Prefer-fixed sections of wild-type **a**,** c**, **e**, **g** and rod-cre **b**, **d**, **f**, **h** mouse retinas were subjected to immunofluorescence with anti-PKM1 **a**, **b** and anti-PKM2 **c**, **d**, anti-opsin **a**–**d**, anti-PDE6β **e**, **f**, and anti-Cre **e**, **f** antibodies. **a**, **b** Merged images of PKM1 and opsin. **c**, **d** Merged images of PKM2 and opsin. **e**, **f** Merged images of PDE6β and Cre. **g**, **h** Omission of primary antibodies. GCL, ganglion cell layer; INL, inner nuclear layer; IPL, inner plexiform layer; ONL, outer nuclear layer; OPL, outer plexiform layer; RIS, rod inner segments; ROS, rod outer segments. Scale bar = 50 μm

In the present study, we used a cre-driver driven by a rod opsin promoter^13^ and examined whether the expressed cre has any effect on PKM1 and PKM2 expression. The results indicate that cre is expressed in rod-cre mice and is absent from wild-type mice (Fig. 3E,F). The expression of PKM1, PKM2, opsin, and PDE6β is indistinguishable in wild-type and rod-cre mice (Fig. 3A–F). These findings suggest that rod-cre has no effect on wild-type mouse rod photoreceptors, but is specific in deleting the floxed exon 10 in the *PKM* gene.

### Effect of loss of PKM2 on cone photoreceptor cells

**Fig. 4:**
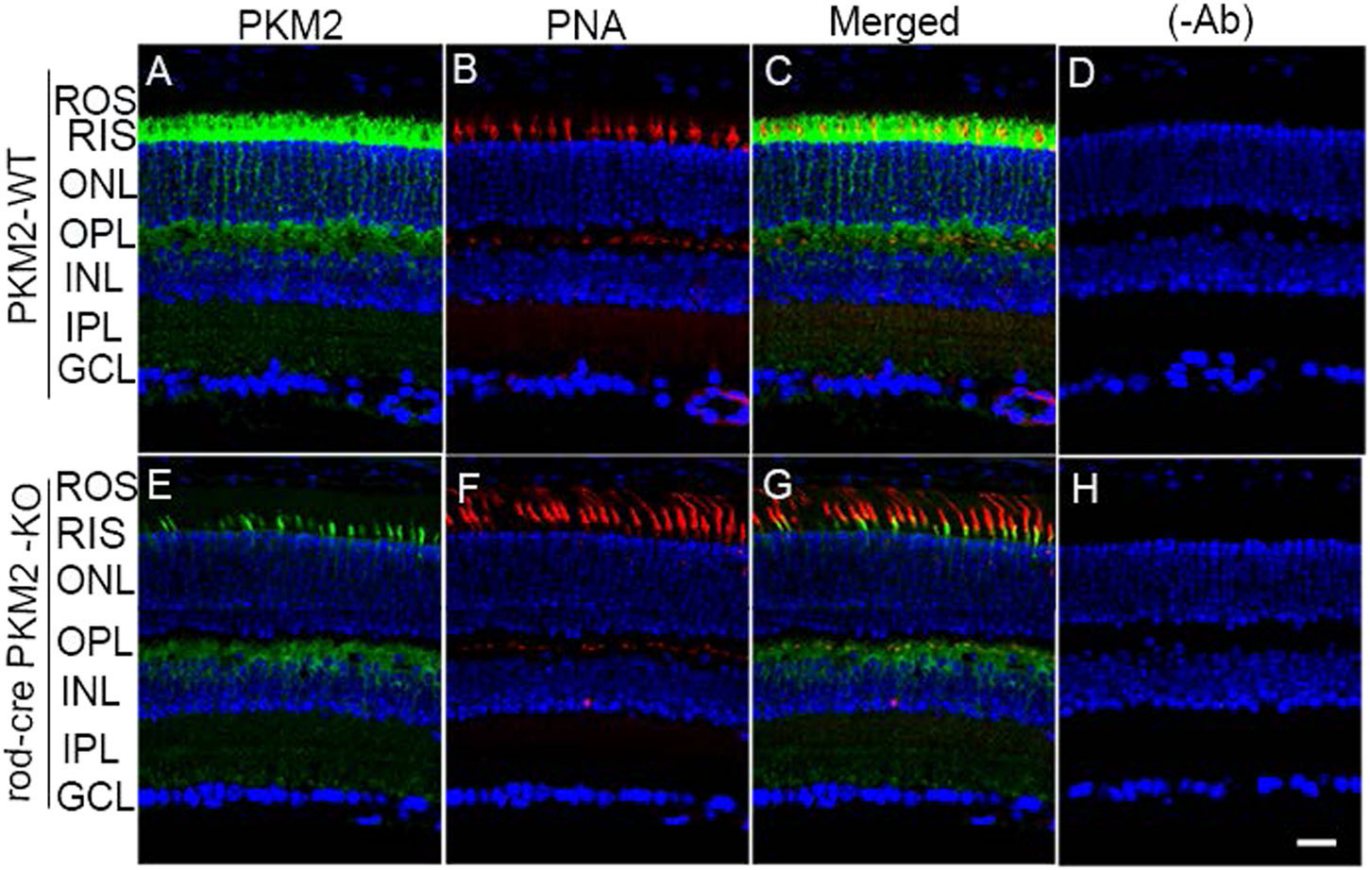
Cone photoreceptor integrity in rod-cre PKM2 KO mice. Prefer-fixed sections of wild-type **a**-** d** and rod-cre PKM2-KO **e**–**h** mouse retinas were subjected to immunofluorescence with anti-PKM2 **a**,** e** and PNA **b**,** f**. **c**, **g** Merged images of PKM2 and PNA. **d**, **h** Omission of primary antibody. GCL, ganglion cell layer; INL, inner nuclear layer; IPL, inner plexiform layer; ONL, outer nuclear layer; OPL, outer plexiform layer; ROS, rod outer segments; RIS, rod inner segment. Scale bar = 50 μm

Retinal sections from PKM2 wild-type and rod-cre PKM2-KO mice were stained or co-stained with PKM2 and peanut agglutinin (PNA), which labels cone OS segments. The co-labeling of PNA and PKM2 in the rod-cre PKM2-KO mouse retina clearly showed that PKM2 was expressed in the cone inner segments (Fig. 4G). We also found PKM2 expression in the OSs of some cone cells (yellow signal, Fig. 4G). The yellow signal observed in the OPL layer of rod-cre PKM2-KO mice could be from cone terminals. Our data suggest that PKM2 loss in rods has no effect on cone structure and density.

### Metabolic phenotype of PKM2 deficiency and upregulation of PKM1

**Fig. 5:**
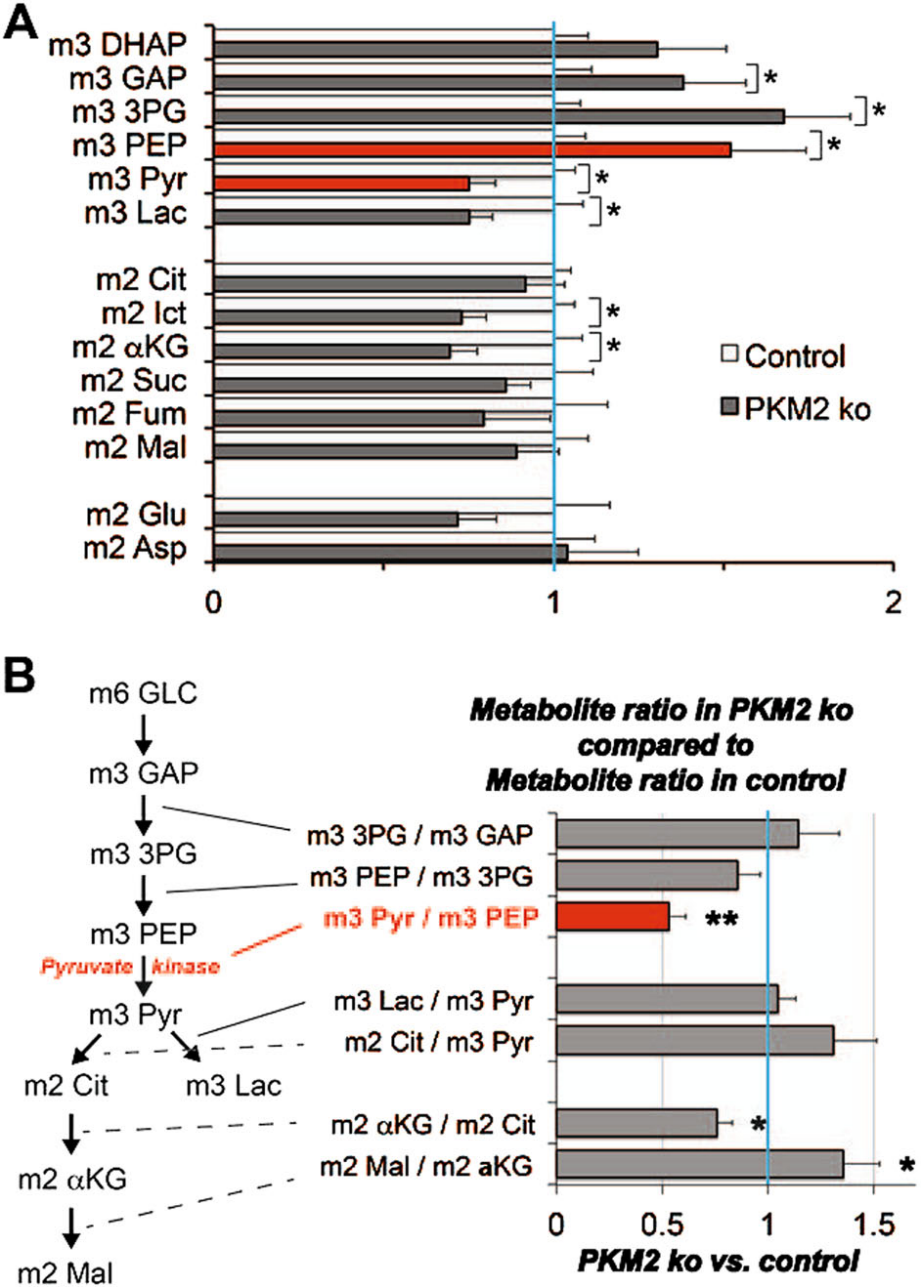
The effect of rod-specific PKM2-deficiency and PKM1 upregulation on metabolic flux in mouse retinas. Mouse retinas were isolated and incubated with 5 mM U-^13^C glucose for 5 min. Metabolites were extracted and analyzed by GC–MS. **a** Comparison of the amounts of labeled metabolites in PKM2-KO vs. control retinas shows that glycolytic intermediates accumulated before the reaction catalyzed by pyruvate kinase. **b** Comparison of the ratios of substrates and products at various points in glycolysis and the TCA cycle shows that only the step catalyzed by pyruvate kinase is significantly affected in the rod-specific PKM2-KO retinas. Data are mean ± SEM (*n *=  8). **p < 0.001, *p < 0.05

A previous study^15^ used purified proteins and specific antibodies to estimate that there are ~ 150 pmols of PKM2 and ~ 26 pmols of PKM1 in a mouse retina. Here we show that blocking PKM2 expression in rods causes a threefold increase in overall expression of PKM1. Assuming that all of the increase occurs in rods, the total amount of PKM1 in rods in the PKM2 KO retinas is  65 pmols, substantially less than the ~ 150 pmols of PKM2 in normal rods. To determine the metabolic consequences of this reduced amount of PK in rods we measured metabolic flux. Isolated retinas were incubated with 5 mM U-^13^C glucose for 5 min, after which they were homogenized and their metabolites extracted and quantified by gas chromatography–mass spectrometry (GC–MS). By 5 min, 57% of the lactate, 35% of the citrate, and 8% of the α-ketoglutarate in the control retinas had been replaced by ^13^C-labeled metabolites. Figure 5A compares the amounts of labeled metabolites at 5 min in PKM2-KO retinas vs. control retinas. ^13^C-labeled glycolytic intermediates before the reaction catalyzed by pyruvate kinase (PK) accumulated to about 150% of normal levels. In Fig. 5B we compare the ratios of the amounts of labeled precursors and products at several points in glycolysis and in the tricarboxylic acid (TCA) cycle. Only the conversion of PEP to pyruvate is significantly slower in the PKM2-KO retinas than in controls. These results show that even though PKM1 is upregulated in the PKM2-KO rods, there is less PK activity and more accumulation of glycolytic intermediates than normal.

### Functional characterization of rods in rod-cre PKM2-KO mice

**Fig. 6:**
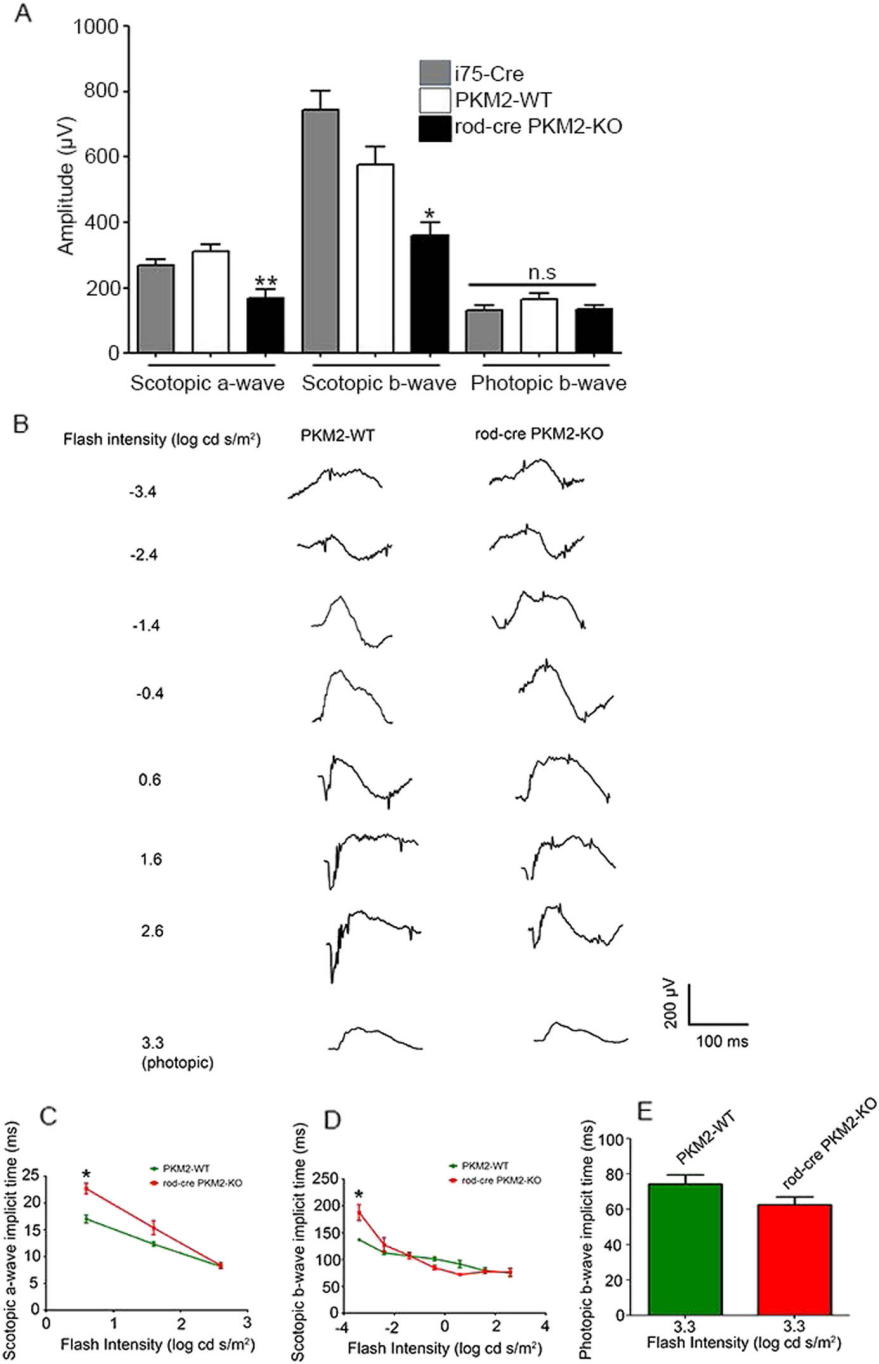
Function of rod-cre PKM2-KO mouse retina. Scotopic a-wave, scotopic b-wave, and photopic b-wave electroretinographic analysis of retinas from 5-month-old i75-Cre, wild-type (PKM2-WT), and rod-cre PKM2-KO mice **a**. Scotopic a-and b-wave amplitudes were measured at a flash intensity of 2.6 log cd s/m^2^, whereas photopic b-wave amplitude was measured at a flash intensity of 3.3 log cd s/m^2^. Data are mean ± SEM (*n *= 6). **p < 0.0022, *p < 0.0138. Representative raw ERG traces recorded from 5-month-old PKM2-WT and rod-cre PKM2-KO mice **b**. Isolated rod ERG measured with a stimulus below the operative range of cones at flash intensities of − 3.4, − 2.4, − 1.4, − 0.4, 0.6, 1.6, and 2.6 log cd s/m^2^. Isolated cone ERG measured at a flash intensity of 3.3 log cd s/m^2^, at which rods were saturated. Scotopic a-wave, scotopic b-wave, and photopic b-wave implicit time intensity-response for PKM2-WT and rod-cre PKM2-KO mice **c-e**. Implicit times for scotopic a-wave were calculated at flash intensities of 0.6, 1.6, and 2.6 log cd s/m^2^. Implicit times for scotopic b-wave were calculated at flash intensities of − 3.4, − 2.4, − 1.4, − 0.4, 0.6, 1.6, and 2.6 log cd s/m^2^. Implicit times for photopic b-wave was calculated at a flash intensity of 3.3 log cd s/m^2^. Data are mean ± SEM (*n *= 6). *p < 0.001

Electroretinography (ERG) data show that there was no significant difference in scotopic a-wave, scotopic b-wave, or photopic b-wave amplitudes between wild-type and rod-cre PKM2-KO mice at 2 months of age (Fig. S1). However, at 5 months of age, there was a significant decrease in scotopic a-wave and scotopic b-wave amplitudes in rod-cre PKM2-KO mice compared with wild-type mice (Fig. 6A), but no significant difference in photopic b-wave amplitudes (Fig. 6A). The ERG wave forms of PKM2-WT and rod-cre PKM2-KO mice are presented in Fig. 6B. At dim flash (− 3.4 log cd s/m^2^), scotopic a- and b-wave amplitudes indicate that rod-cre PKM2 KO mice has significantly increased implicit times compared with PKM2-WT; at bright flash, the implicit times of scotopic a- and b-, and photopic b-wave amplitudes are almost identical in these two genotypes (Fig. 6C-E). There was no significant difference in either rod or cone function between wild-type and i75-Cre mice (Fig. 6A), suggesting that Cre-recombinase has no adverse effect on the function of the retina. These results suggest that PKM2 is important for rod photoreceptor function.

### Morphological characterization of rod-cre PKM2-KO mice

**Fig. 7:**
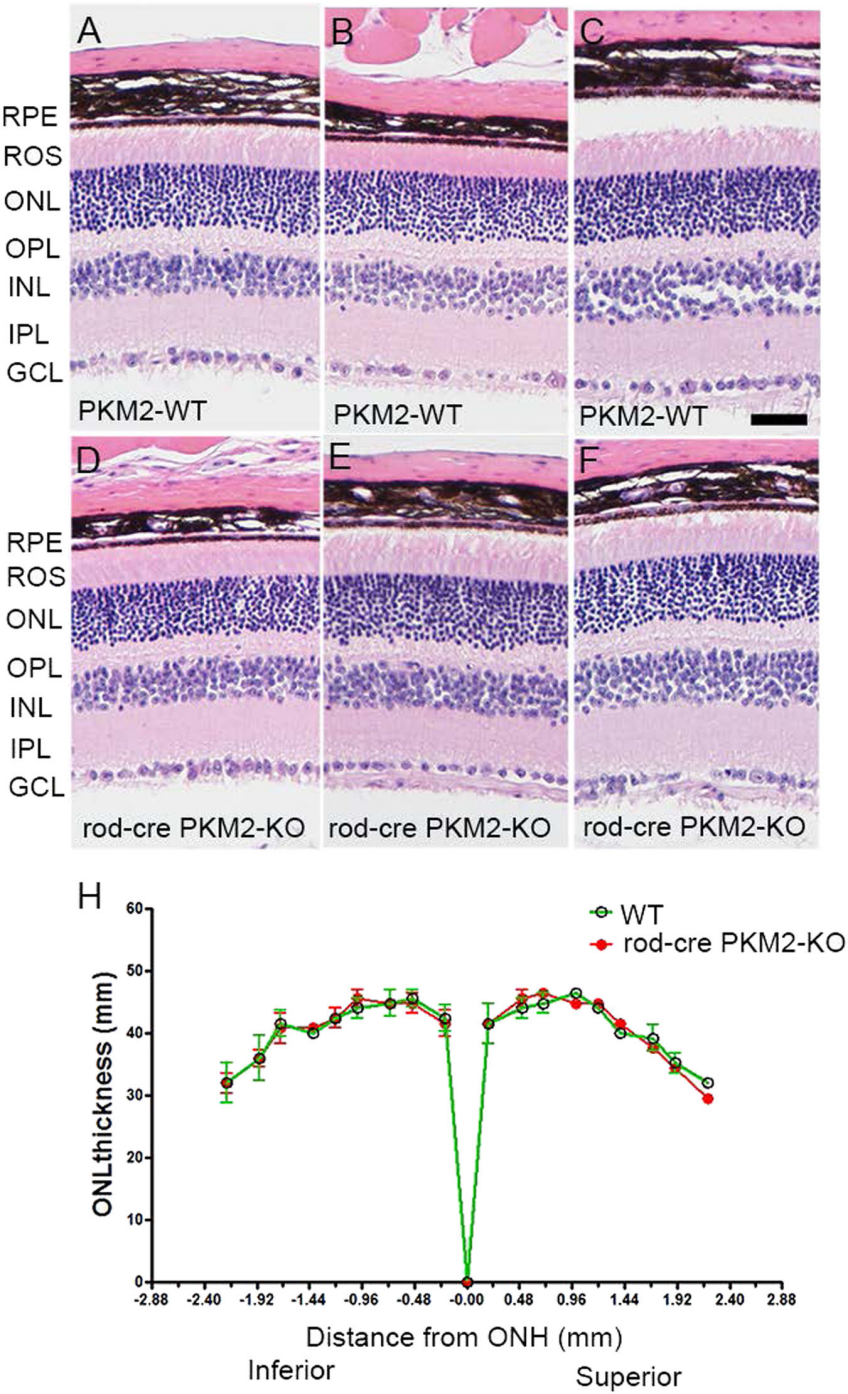
Morphology of rod-cre PKM2-KO retina and assessment of rod outer segment integrity. Morphologic examination of three independent mouse retinas from 5-month-old wild-type **a**–**c** and rod-cre PKM2-KO mice **d**–**f**. GCL, ganglion cell layer; INL, inner nuclear layer; IPL, inner plexiform layer; ONL, outer nuclear layer; OPL, outer plexiform layer; RPE, retinal pigment epithelium; ROS, rod outer segments. Scale bar = 50 μm. Quantification of morphological changes **h**. Plots of total retinal thickness in the inferior and superior regions of the retinas of wild-type, and rod-cre PKM2-KO mice are shown. Values are mean ± SEM (*n* = 3). ONH, optic nerve head

**Fig. 8:**
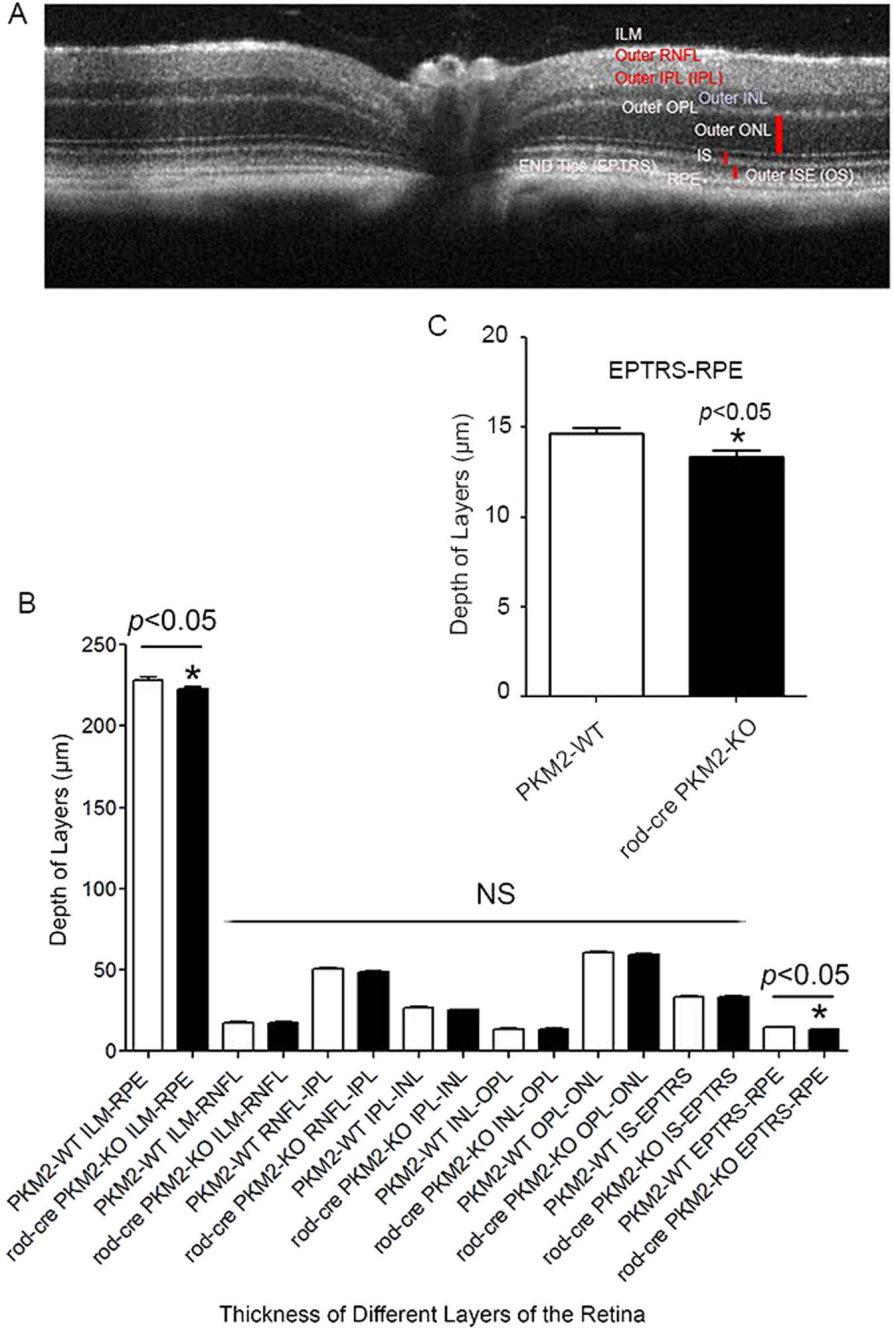
Morphological architecture of retina using optical coherence tomography (OCT). Retinal thickness of each layer from wild-type and rod-cre PKM2-KO mice was assessed by OCT. **a** Morphological architecture of the retina, showing individual layers. These layers include the inner limiting membrane (ILM), outer retinal nerve fiber layer (RNFL), outer inner plexiform layer (IPL), outer inner nuclear layer (INL), outer plexiform layer (OPL), outer nuclear layer (ONL), inner segment ellipsoid (IS), outer segment ellipsoid (OS), end tips (EPTRS), and retinal pigment epithelium (RPE). **b** Quantitative OCT analysis of the depth of each retinal layer of wild-type and rod-cre PKM2-KO mice. Inset **c** thickness between EPTRS and RPE in wild-type and rod-cre PKM2-KO mice. Data are mean ± SEM (*n* = 14). *p < 0.05

To determine the effect of PKM2 loss on retinal structure, eye sections from 2- and 5-month-old PKM2 wild-type and rod-cre PKM2-KO mice were stained with hematoxylin and eosin (H&E) and the morphology was examined with light microscopy. There was no difference in the retinal structures of wild-type and rod-cre PKM2-KO mice, and the ROS segments appeared to be well organized in 2- (data not shown) and 5-month-old mice (Fig. 7A–F). Quantitative analysis of the superior and inferior regions of the outer nuclear layer (ONL) layer of the two groups of animals showed no significant differences in the average ONL thickness measured at 0.24 mm intervals from the optic nerve head to the inferior and superior ora serrata (Fig. 7H), indicating that there was no difference in rod photoreceptor viability in wild-type and rod-cre PKM2 KO mice. As the morphological observation of H&E staining is qualitative, we conducted optical coherence tomography (OCT) on 5-month-old live mice to examine retinal layer thickness. Our analysis indicated that the thickness from inner limiting membrane to retinal pigment epithelium (RPE) and thickness from end tips to RPE are significantly reduced in rod-cre PKM2-KO mouse retinas compared with wild-type retinas (Fig. 8A,B,C). There was no significant difference in the thickness of other layers in wild-type and rod-cre PKM2-KO mice (Fig. 8B). Collectively, our results show that loss of PKM2 reduced the thickness of the photoreceptor end tips. Our data suggest that PKM2 is important for the maintenance of photoreceptor structure.

### Photoreceptor-specific protein expression in rods from rod-cre PKM2-KO mice

**Fig. 9:**
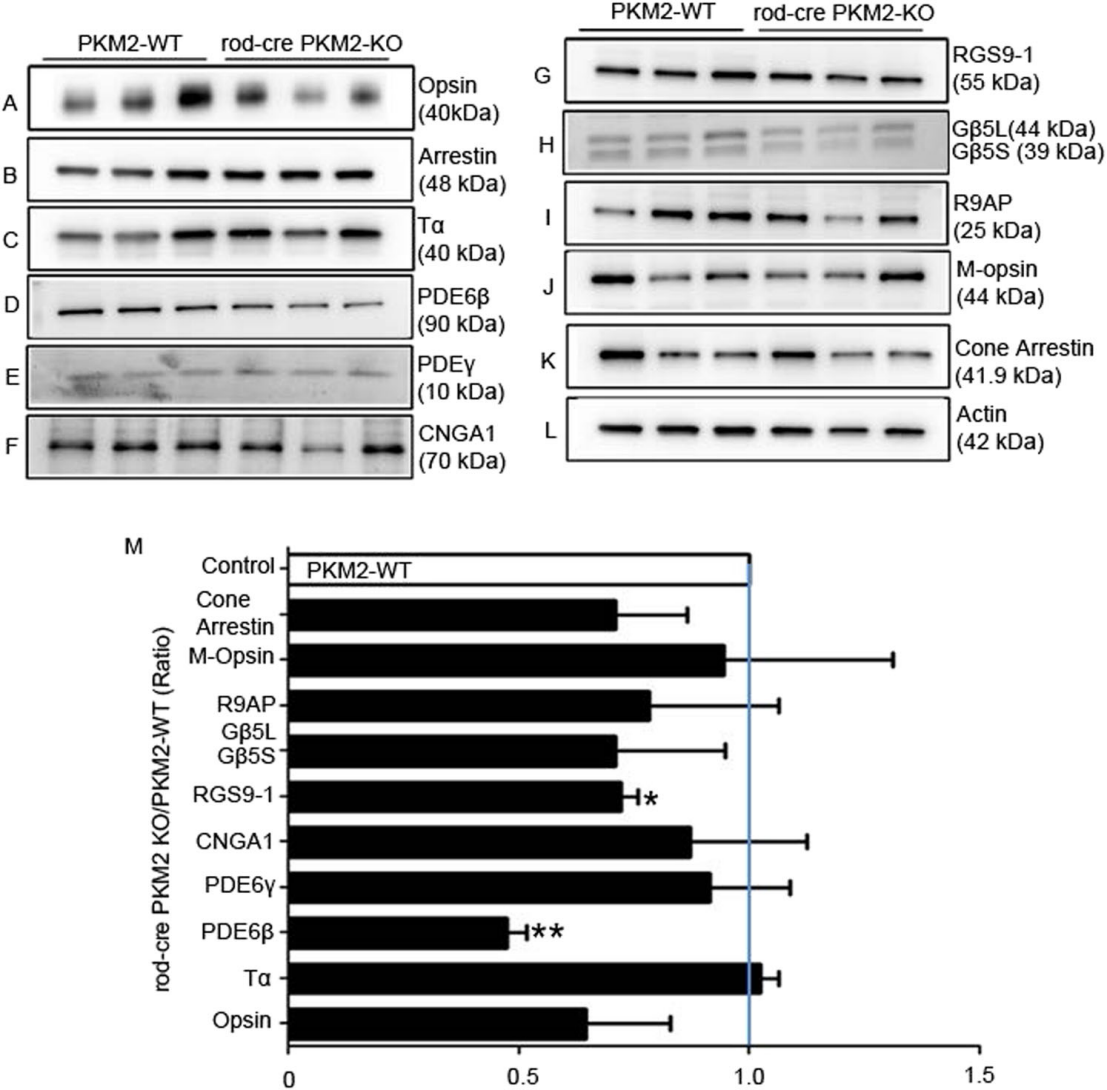
Expression levels of photoreceptor-specific proteins in wild-type and rod-cre PKM2-KO mice. Retinal homogenates (5.0 µg protein) from three independent wild-type and rod-cre PKM2 KO mice were subjected to immunoblot analysis with anti-opsin **a**, anti-arrestin **b**, anti-Tα **c**, anti-PDE6β **d**, anti-PDEγ **e**, anti-CNGA1 **f**, anti-RGS9-1 **g**, anti-Gβ5L/S **h**, anti-R9AP **i**, anti-M-opsin **j**, anti-cone-arrestin **k**, and anti-actin **l** antibodies. We normalized the protein expression to rod arrestin **m** and then calculated the ratios (rod-cre PKM2-KO/PKM2-WT). Data presented as a ratio are mean ± SEM (*n = *3). **p<0.01, *p < 0.02

**Fig. 10:**
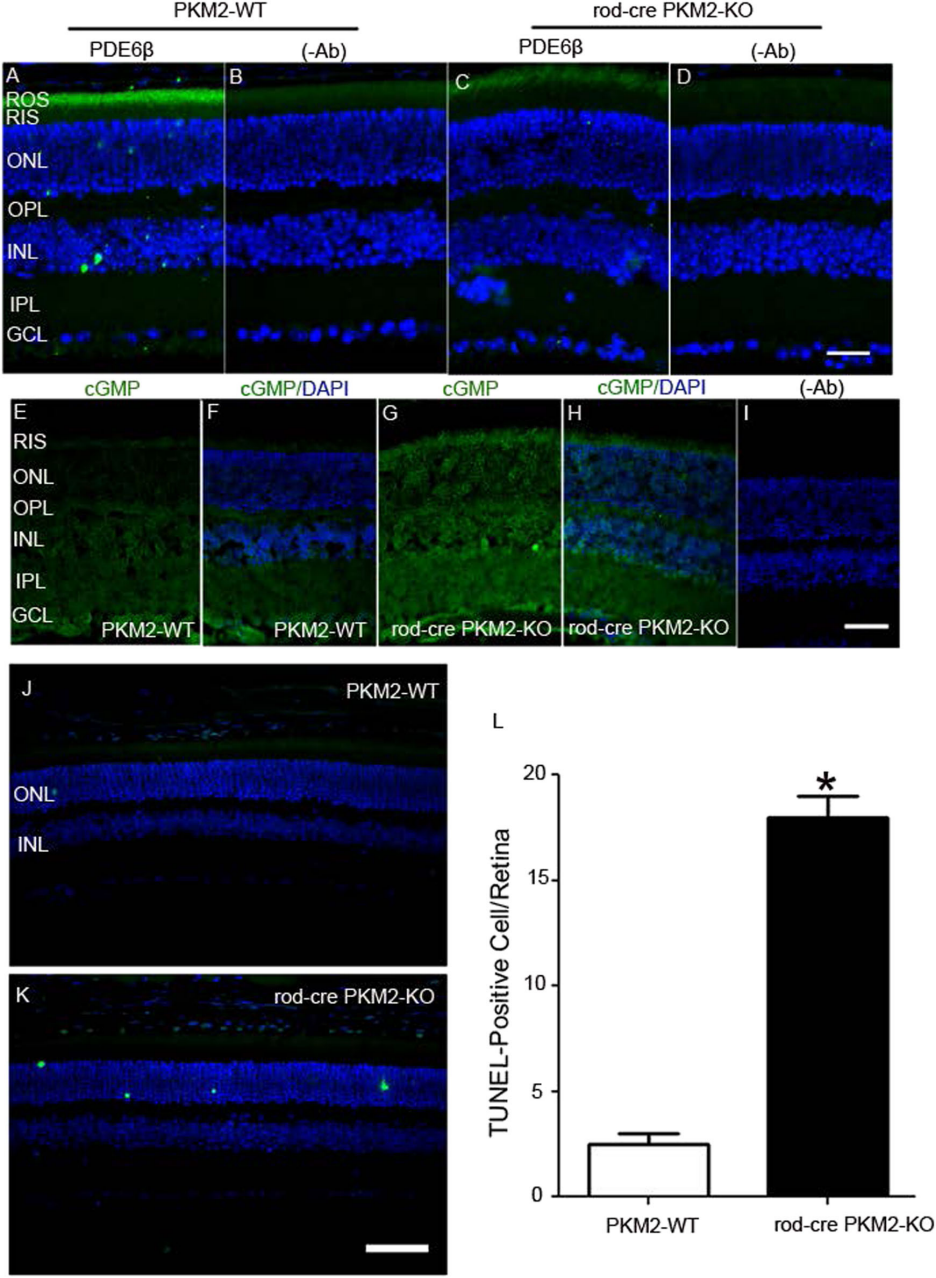
Decreased PED6 β, increased cGMP, and increased TUNEL staining in rod-cre PKM2 KO mouse retinas. Prefer-fixed sections of wild-type **a** and rod-cre PKM2-KO **c** mouse retinas were subjected to immunofluorescence with anti-PDE6β **a**,** c** antibody. **b**, **d** Omission of primary antibody. GCL, ganglion cell layer; INL, inner nuclear layer; IPL, inner plexiform layer; ONL, outer nuclear layer; OPL, outer plexiform layer; RIS, rod inner segments; ROS, rod outer segments. Scale bar = 50 μm. Cryosections of wild-type **e**,** f** and rod-cre PKM2-KO **g**,** h** mouse retinas were subjected to immunofluorescence with anti-cGMP **e**–**h** antibody. **f**, **h** cGMP (green)/DAPI (blue) staining. **i** Omission of primary antibody. Prefer-fixed retinal sections of PKM2-WT and rod-cre PKM2-KO mice were examined for cell death with in situ localization of apoptosis using TUNEL **j**,** k**. TUNEL-positive cells were counted on the entire retina **l**. Data are mean ± SEM, (*n* = 3). *p < 0.001

Our structural and functional analysis of rod-cre PKM2-KO mice suggests that there might be a change in photoreceptor protein expression. We carried out immunoblot analysis on retinal lysates prepared from 5-month-old mice with rod opsin, rod arrestin, rod-Trα, PDE6β, PDEγ, cyclic nucleotide gated channel ɑ-subunit (CNGA1), RGS9-1, Gβ5L, Gβ5S, R9AP, M-opsin, cone-arrestin, and actin antibodies (Fig. 9A–L). We normalized the protein expression to rod arrestin and then calculated the ratios (rod-cre PKM2-KO/PKM2-WT) (Fig. 9M). Our results revealed generally lower expression of most photoreceptor proteins. Several of the decreases were statistically significant including rod PDE6β and RGS9-1 levels in rod-cre PKM2-KO mice compared with wild-type mice (Fig. 9D,G,M). We performed immunohistochemistry with a rod PDE6β antibody and confirmed reduced expression of PDE6β in the OSs of rod-cre PKM2-KO mice (Fig. 10A,C). PDE6β is the effector enzyme in the vertebrate visual transduction cascade; activated PDE6 hydrolyzes intracellular cGMP, resulting in a closure of cGMP-gated channels and hyperpolarization of the photoreceptor plasma membrane^16^. We hypothesized that decreased PDE6β enzyme may affect the hydrolysis of cGMP, and found no excessive cGMP accumulation in wild-type photoreceptors, whereas cGMP levels were highly elevated in rod-cre PKM2-KO mouse retinas (Fig. 10E–H). We used two cGMP antibodies, a rabbit polyclonal antibody from Millipore (Billerica, MA) (Fig. S3) and a sheep anti-cGMP antibody obtained from Dr Jan De Vente (Maastricht, The Netherlands) (Fig. 10E–H), and both antibodies show similar results.

Increased levels of cGMP have been shown to induce photoreceptor degeneration^17–20^; therefore, we examined whether loss of PKM2 induces cell death in rod photoreceptor cells of rod-cre PKM2-KO mice using in situ terminal deoxynucleotidyl transferase (TdT) dUTP nick-end labeling (TUNEL) staining to localize apoptosis (Fig. 10J,K) and counted the number of TUNEL-positive cells over the entire retina (Fig. 10L). Our results show significantly increased cell death in the ONL of rod photoreceptor cells of rod-cre PKM2-KO mice compared with PKM2-WT mice. These observations suggest that rod photoreceptors require PKM2 to stay alive.

### PKM2 regulates the pde6β promoter activity

**Fig. 11:**
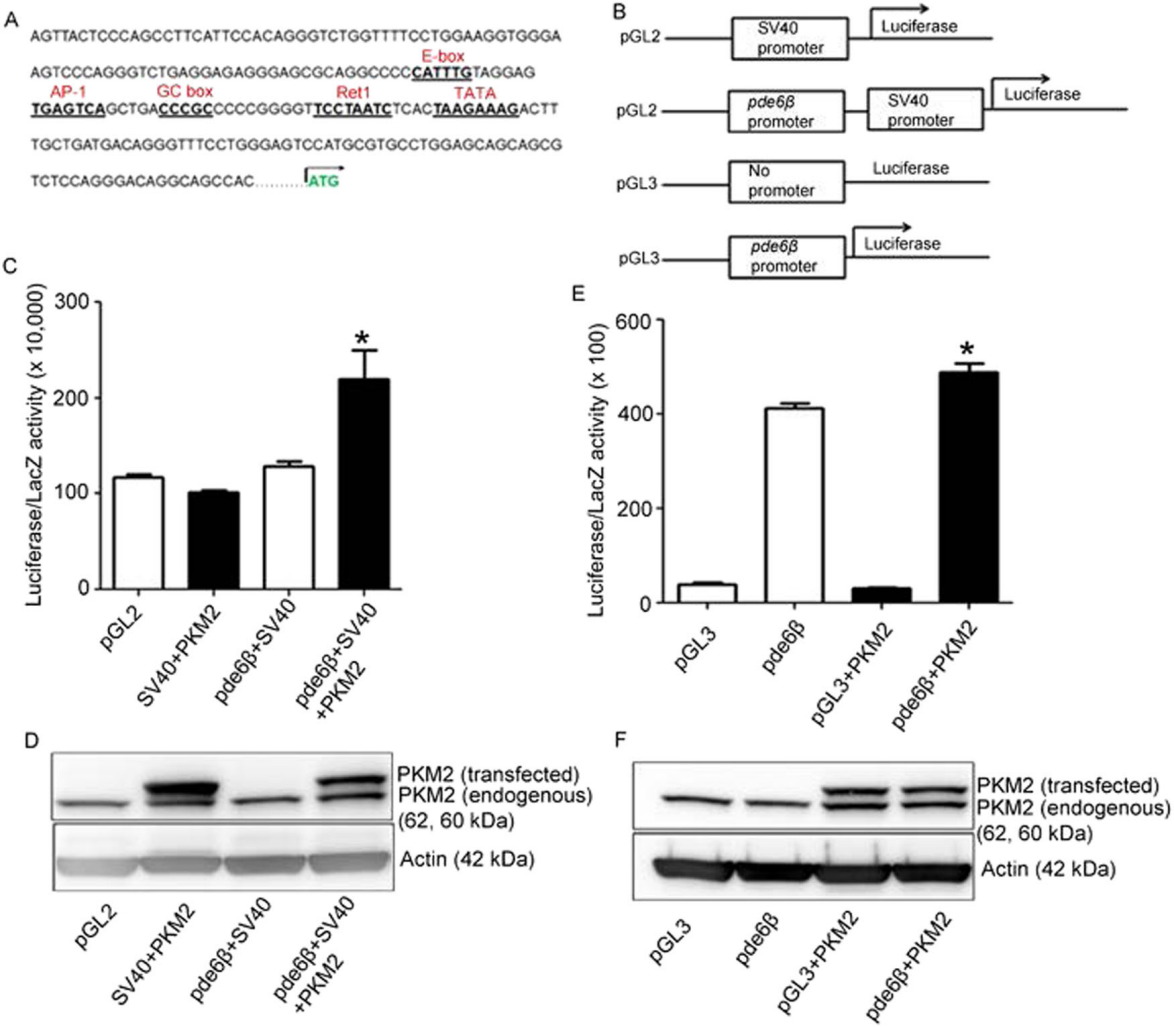
PKM2 regulates pde6β promoter activity in vitro: Sequence of the human pde6β proximal promoter region showing the potential regulatory elements: E box, AP-1, GC box, Ret1, and TATA box are labeled **a**. The regulatory region constructs used in this study are: pGL2-SV40-luciferase, pGL2-pde6β-SV40-luciferase, pGL3-luciferase and pGL3- pde6β-luciferase **b**. Empty vectors or promoter constructs were transfected into HEK-293T cells in the presence of β-galactosidase, and either flag-tagged PKM2 or absence of flag-tagged PKM2. Forty-eight hours later, cells were lysed and the luciferase and β-galactosidase activity were measured, and the luciferase activity was normalized to β-galactosidase activity **c**, **e**. Remaining lysates were used for immunoblot analysis with Flag (transfected), PKM2 (endogenous) and actin antibodies **d**, **f**. Data are mean ± SEM, (*n* = 3). *p < 0.05

To determine whether PKM2 regulates expression of PDE6β, empty or pde6β-SV40 promoter, or pde6β promoter (Fig. 11A,B) vectors were transfected into HEK-293T cells by calcium phosphate method with a vector carrying β-galactosidase with or without Flag-tagged PKM2 constructs. Forty-eight hours later, cells were collected and measured for luciferase and β-galactosidase activities and normalized the luciferase activity to β-galactosidase activity. We found that PKM2 has no effect on SV40-promoter; however, addition of pde6β promoter region to SV40 promoter exhibits a significantly enhanced luciferase activity in the presence of PKM2 (Fig. 11C). We also found significantly increased pde6β-promoter activity in the presence of PKM2 (Fig. 11E). The expression levels of PKM2 are comparable between pde6β-SV40 and pde6β promoter transfections (Fig. 11D,F). We found a 25-fold higher activation of SV40 promoter in HEK-293T cells compared with pde6β promoter (Fig. 11C,E). The higher promoter activity in pde6β alone could be due to endogenous PKM2 in HEK-293T cells (Fig. 11E,F). Collectively, these results suggest that PKM2 regulates the pde6β promoter activity.

### Effect of PKM2 loss on glucose transporter-1

**Fig. 12:**
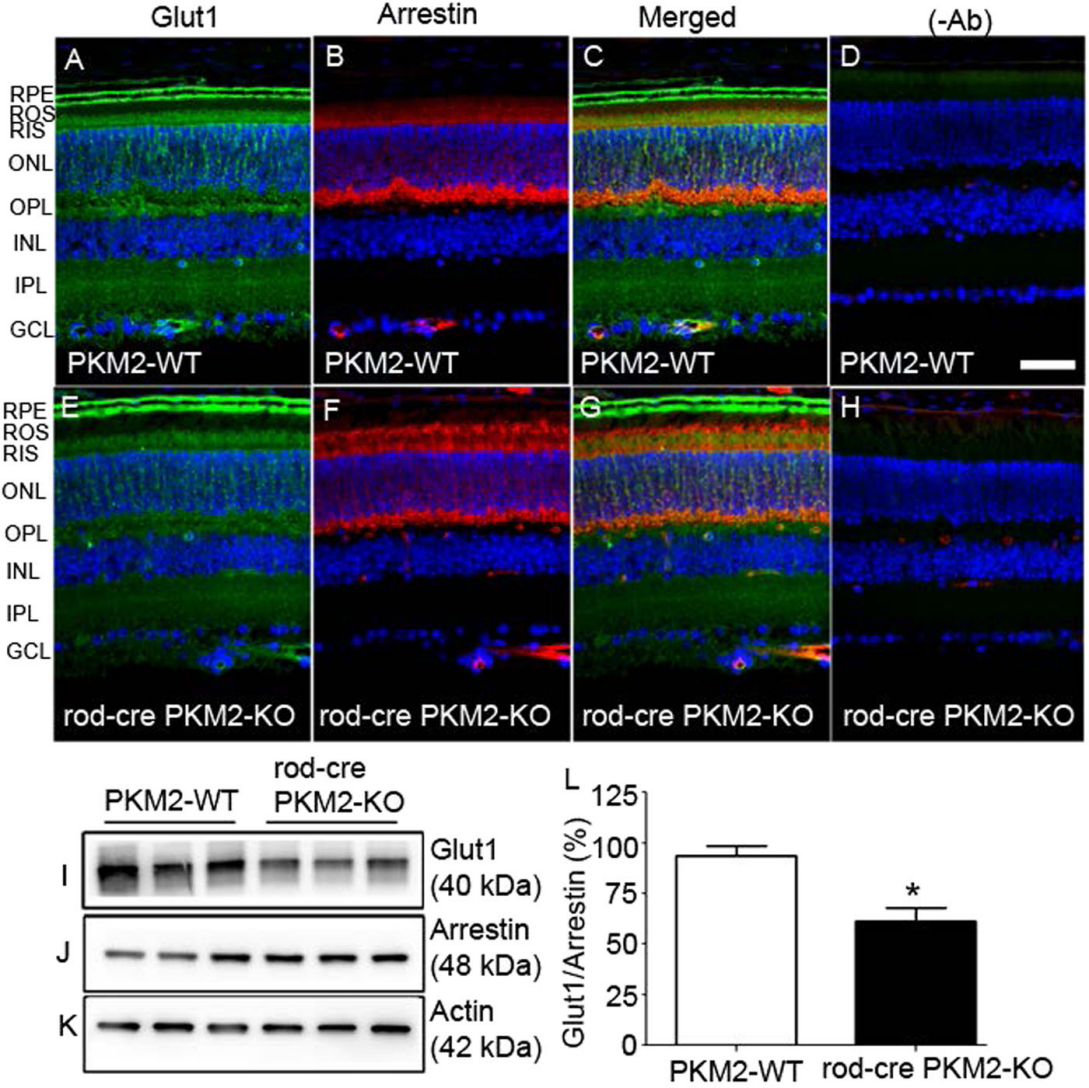
Effect of PKM2 loss on glucose transporter Glut1 expression in the retina. Prefer-fixed sections of wild-type **a**-** d** and rod-cre PKM2-KO **e**–**h** mouse retinas were subjected to immunofluorescence with anti-Glut1 **a**,** c**,** e**,** g** and anti-arrestin **b**,** c**,** f**,** g** antibodies. **c**, **g** Merged images of Glut1 and arrestin. **d**, **h** Omission of primary antibodies. GCL, ganglion cell layer; INL, inner nuclear layer; IPL, inner plexiform layer; ONL, outer nuclear layer; OPL, outer plexiform layer; RIS, rod inner segments; RPE, retinal pigment epithelium; ROS, rod outer segments. Scale bar = 50 μm. Retinal proteins prepared from three independent wild-type and rod-cre PKM2-KO mice were subjected to immunoblot analysis with anti-Glut1 **i**, anti-arrestin **j**, and anti-actin **k** antibodies. Densitometric analysis of immunoblots was performed in the linear range of detection. Absolute values were then normalized to arrestin **l**. Data are mean ± SEM (*n *= 3). *p < 0.01. The normalized PKM2 wild-type control was set as 100%

It was previously shown that reduced glucose decreases the visual function in animals^7^. Therefore, we examined whether loss of PKM2 in rods affects the major glucose transporter, Glut1, in rod photoreceptor cells. Our results revealed the expression of Glut1 in RPE, inner segments, and the outer plexiform layer in both wild-type and rod-cre PKM2-KO mice (Fig. 12A,E). The immunohistochemical results indicated that there is reduced Glut1 immunoreactivity in 5-month old rod-cre PKM2-KO mice compared with wild-type mice (Fig. 12A,E). Immunoblot analysis confirmed the significantly decreased levels of Glut1 in rod-cre PKM2-KO mice compared to wild-type mice (Fig. 12I–L). These observations suggest that loss of PKM2 reduces expression of Glut1 in rod photoreceptor cells.

### Effect of PKM2 loss on lipid biochemistry

**Fig. 13:**
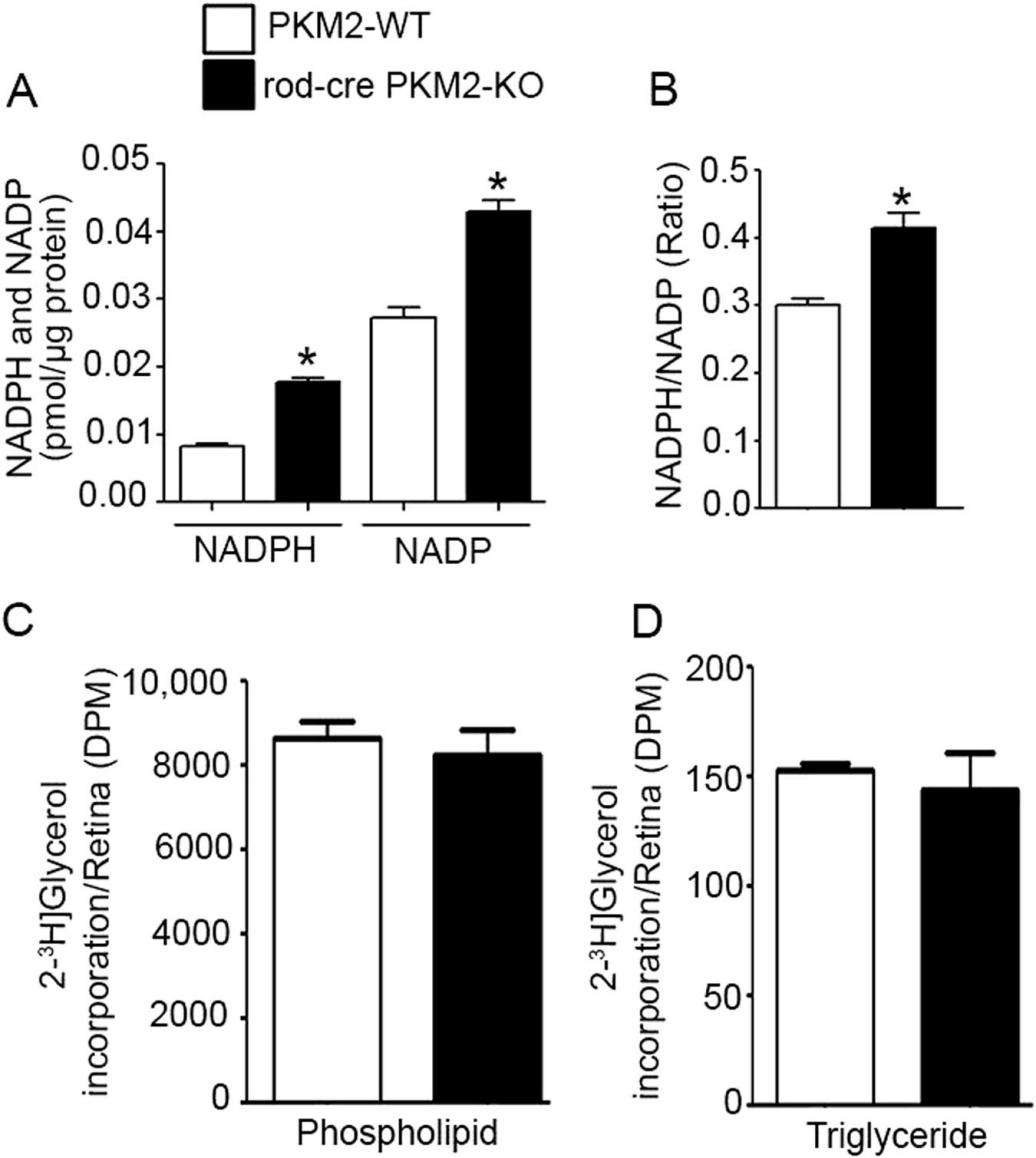
Determination of NADPH and NADP levels in wild-type and rod-cre PKM2-KO mouse retinas. Mouse retinal tissues from wild-type and rod-cre PKM2-KO mice were used to measure NADPH and NADP levels **a** and their ratio was presented **b**. Data are mean ± SEM (*n *= 3). *p < 0.05. PKM2-WT and rod-cre PKM2-KO mouse retinas were placed in Ringer solution and incubated in the presence of radiolabeled [2-^3^H] glycerol for 45 min at room temperature. Total lipids were extricated and subjected to TLC to separate phospholipids **c** and triglycerides **d** followed by counting the radioactivity. Data mean ± SEM (*n* = 4)

The NADPH generated through the PPP system is pivotal for lipid synthesis and antioxidant metabolism. We examined whether loss of PKM2 affects NADPH generation, and found significantly increased NADPH levels in rod-cre PKM2 KO mouse retinas compared with wild type (Fig. 13A). The NADPH/NADP ratio was significantly higher in rod-cre PKM2-KO mouse retinas than in wild-type mouse retinas (Fig. 13B). To test whether the increased NADPH favors increased lipid synthesis in rod-cre PKM2 KO mice, we carried out the isotope labeling with [2-^3^H] glycerol and determined the incorporation of labeled glycerol into the newly synthesized phospholipids and triglycerides resolved by thin layer chromatography followed by counting the radioactivity for quantification. Our results show no significant difference between rod-cre PKM2 KO mice and wild-type mice in the biosynthesis of phospholipid (Fig. 13C) and triglycerides (Fig. 13D). These studies suggest that increased NADPH does not favor lipid biosynthesis in rod-cre PKM2 KO mice. Quantification of lipid molecular species of the three most abundant phospholipids (phosphatidylcholine (PC), phosphatidylethanolamine (PE), and phosphatidylserine (PS)) was performed using a triple quadrupole mass spectrometer. Wild-type and rod-cre PKM2-KO mouse retinas had no remarkable differences in ROS PC, PE, and PS lipid molecular species (Fig. S3). However, the level of PC 38 : 06 was slightly (but significantly) lower in rod-cre PKM2-KO mouse retina compared with wild-type retina (Fig. S3A). Furthermore, PS 40 : 06 levels were slightly (but significantly) higher in rod-cre PKM2-KO retina than in wild-type retina (Fig. S3C). These differences in PC and PS lipid species may not have any biological significance in vivo.

## Discussion

Photoreceptors are non-proliferating cells, yet predominantly express PKM2 and express little PKM1^6^. We found that loss of PKM2 significantly upregulates PKM1 in rod photoreceptor cells and this upregulated PKM1 might complement or substitute for the functions of PKM2 in vivo. Recent studies have also observed the upregulation of PKM1 in the absence of PKM2^21,22^. However, our metabolic flux analysis shows that 2.5-fold overexpression of PKM1 in mouse rods does not provide the same catalytic power as the normal amount of PKM2 in mouse rods. The accumulation of glycolytic intermediates in the absence of PKM2 could mean that PKM1 has a higher *K*_m_ for PEP in vivo or that there is a lower overall level of PK activity. The significant decline in rod function at 5 months indicates the normal levels PKM2 are essential. Morphological characterization showed a significant decrease in the overall retinal thickness in rod-cre PKM2-KO mice, especially the photoreceptor tips, compared to wild-type mice. There was also photoreceptor cell death in these retinas determined by TUNEL assay. Consistent with our results, knockdown of PKM2 specifically in mouse rod photoreceptors reduces the overall length of photoreceptors^21^. It has been shown previously that glucose deprivation affects the ERG in superfused, oxygenated rat retina^7^. PKM2 has been previously shown to regulate the Glut1 expression^23^, and in the absence of PKM2 we found decreased expression of Glut1 in photoreceptors. We also found that glycolytic intermediates accumulate and that there is a slightly reduced flux of carbons from glucose through TCA cycle intermediates. The metabolic bottleneck at the pyruvate kinase step in rods may contribute to the decline in the ERG responses in rod-cre PKM2-KO mice. The consequences of the ERG reduction could be caused by reduced expression of photoreceptor proteins including proteins in the phototransduction cascade, the shortening of the OSs, or the loss of rod photoreceptors. Interestingly, PDE6β promoter activity assays indicate that PKM2 may regulate PDE6β expression in vivo. The earlier published studies, along with our present findings, suggest that PKM2 may regulate the expression of PDE6β as a transcriptional co-activator in photoreceptor cells.

Our studies show that PKM1 is a minor protein in rods, and its threefold upregulation in the absence of PKM2 may alter photoreceptor function. A recent study showed that combined shRNA-mediated knockdown of PKM1/PKM2 resulted in the shortening of OS segments and this phenotype could be reversed with a subretinal administration of a cDNA encoding PKM2, but not PKM1^21^. However, forced expression of PKM1 in the presence of endogenous PKM2 reduced the length of the OS^21^. This situation does not exist in vivo. Although we do not have the data on the functional role of PKM1 in vivo due to unavailability of floxed PKM1mice, the present study still provides important information that PKM1 cannot substitute for PKM2 in photoreceptor functions.

It has been suggested that the inactive form of PKM2 redirects the glucose flux and activates the PPP pathway^4^. In cancer cells, PKM2 activity reduction led to distinctive accumulation of glycolytic intermediates and increased accumulation of NADPH^5^. In the present study, we found a significant increase of NADPH and our glucose flux experiments show an accumulation of glycolytic intermediates in rod-cre PKM2-KO mouse retinas, which may favor the PPP pathway as has been reported in yeast cells^4^. This suggests that there is more NADPH production, which would favor lipid biosynthesis, but we found no effect on lipid biosynthesis. The increased NADPH levels in rod-cre PKM2 KO mice could be used for anti-oxidant metabolism to prevent cell death in these mouse retinas. It is reasonable to hypothesize that accumulation of glycolytic intermediates would make the cells more anabolic and bigger, but in photoreceptors we found that the cells undergo apoptosis and the OSs are shorter. Overall, our findings show that PKM2 has a neuroprotective role in non-dividing photoreceptor cell structure and function.

Our studies show that PKM1 cannot fully complement the functions of PKM2, suggesting that there may be another source/pathway of acetate for TCA cycle production of ATP. What are these possible sources? Although it may be that the up-regulation of PKM1 could produce an adequate amounts of acetate, this is unlikely from the arguments presented above. The other source of 3-carbon compounds such as pyruvate and lactate derived from other cells such as Muller cells. However, current thinking is that photoreceptors make lactate that is shuttled to Muller cells, not vice versa, since Muller cells do not express pyruvate kinase^15^. Generation of acetate by beta oxidation of fatty acids, which occurs in the mitochondria, is another possibility. Joyal et al^24^ recently reported that retinal photoreceptor cells can use fatty acids as well as glucose for ATP production. In this case, there would be an influx of fatty acids from some external source in greater abundance than needed for membrane synthesis and OS renewal. The most obvious source is the RPE. We^25^ and others^26^ established some time ago that the n3 and n6 polyunsaturated fatty acids (PUFAs) that are the major components of ROS membrane phospholipids are recycled from the RPE to the inner segment. Thus, a mechanism exists for movement of large amounts of fatty acids from the RPE to the inner segments of photoreceptor cells. Once inside the cell, the fatty acids could be used for membrane synthesis as well as oxidative production of ATP. We hypothesize that these recycled fatty acids contain some that are taken up from the circulation, so that what returns to the inner segment is actually more than necessary for membrane biosynthesis. The extra fatty acids could then be used to generate acetate to drive the TCA cycle. However, there is selectivity here because we have shown that the n3 PUFA are essential components of ROS membranes^27,28^. If utilized for production of acetate, their content in the ROS would decrease if they or their precursors were removed from the diet. We have shown that does not happen^29^, suggesting that there may be two pools of fatty acids in the inner segments, one containing PUFA that is used for membrane renewal and another that is likely composed of shorter chain saturated and mono-unsaturated fatty acids that are directed to the mitochondria for β-oxidation. The key to understanding how these mice survived is to find the source of energy production in PKM2 knockout mice retinas.

## Materials and methods

### Materials

#### Antibodies

Polyclonal pPKM2 (Y105), PKM2, and PKM1 antibodies were obtained from Cell Signaling (Danvers, MA). Anti-PDEγ and anti-actin antibody were purchased from Affinity BioReagents (Golden, CO). The GAP protein antibodies (RGS9-1, Gβ5_L_, Gβ5_S_, and R9AP) were kindly provided by Dr Theodore G. Wensel (Baylor College of Medicine, Houston, TX). Anti-PDE6β, anti-Trα subunit, and goat secondary antibodies were procured from Santa Cruz Biotechnology (Santa Cruz, CA). Rabbit polyclonal anti-red/green cone opsin (M-opsin), rabbit polyclonal anti-cGMP, anti-cone arrestin, and rabbit and mouse secondary antibodies were obtained from Millipore. Mouse monoclonal anti-Cre antibody suitable for immunohistochemistry was purchased from Abcam (Cambridge, MA). Polyclonal Glut1 antibody was procured from Novus Biologicals (Littleton, CO). Monoclonal 1D4 rhodopsin antibody was a kind gift from Dr James F. McGinnis (University of Oklahoma Health Sciences Center). Monoclonal CNGA1 was kindly provided by Dr Robert S. Molday (University of British Columbia, Canada). Sheep anti-cGMP antibody was provided by Dr Jan De Vente (Maastricht University, Maastricht, The Netherlands).

#### Animals

All animals were treated in accordance with the ARVO Statement for the Use of Animals in Ophthalmic and Vision Research, and the NIH Guide for the Care and Use of Laboratory Animals. The protocols were approved by the Institutional Animal Care and Use Committee at the University of Oklahoma Health Sciences Center. Animals were born and raised in our vivarium and kept under dim cyclic light (40–60 lux, 12 h light/dark cycle). PKM2 floxed mice were purchased from The Jackson Laboratory (Bar Harbor, Maine).

#### Chemicals

The NADP/NADPH quantification kit and In Situ Fluorescein Cell Death Detection Kit were procured from Sigma (St. Louis, MO). All other reagents were of analytical grade and purchased from Sigma.

#### Generation of photoreceptor-specific conditional PKM2 knockout mice

Photoreceptor-specific conditional PKM2 knockout mice (rod-cre PKM2-KO) were prepared with the Cre-lox technique by mating floxed PKM2 animals with mice expressing Cre recombinase (i75-Cre) under the control of the 4 kb mouse opsin promoter^13^. In the presence of Cre recombinase, the floxed exon 10 of the PKM2 allele is deleted. The genotype of the rod-cre PKM2-KO mice (i.e., animals carrying the *cre* transgene and homozygous for the PKM2-floxed allele) was confirmed by PCR analysis of tail DNA. To identify opsin*-cre*, PCR was performed with 1 μl of genomic DNA and sense (5′-TCAGTGCCTGGAGTTGCGCTGTGG-3′) and antisense (5′-CTTAAAGGCCAGGGCCTGCTTGGC-3′) primers to amplify a 500 bp product. To identify PKM2 floxed mice, we used sense (5′-AGG TAG GAG GCG GCA GTG-3′) and antisense (5′-CCA CTC ACT CTT GGC ATC C-3′) primers to amplify genomic DNA by PCR. The wild-type allele generates a 79 bp product and the floxed allele generates a 126 bp product. The wild-type and PKM2-KO mice were on C57Bl6 background. We also screened wild-type and rod-cre PKM2 KO mice for *rd1* and *rd8* mutations, and all these mice were negative for these mutations.

#### Preparation of mouse ROSs

ROSs were prepared according to the method described earlier^30^. Eight retinas from four mice were homogenized in 1.25 ml of ice-cold 47% sucrose solution containing buffer A (100 mM NaCl, 1 mM EDTA, 1 mM phenylmethylsulfonyl fluoride (PMSF), and 10 mM Tris-HCl, pH 7.4). Retinal homogenates were transferred to 4.5 ml centrifuge tubes and sequentially overlaid with 1.5 ml of 37% sucrose and 1.0 ml of 32% sucrose dissolved in buffer A. The tubes were spun at 82,000 × *g* for 1 h at 4 °C. The 32%/37% interfacial sucrose band containing ROS membranes was harvested and diluted with 10 mM Tris-HCl (pH 7.4) containing 100 mM NaCl and 1 mM EDTA, and centrifuged at 27,000 × *g* for 30 min. The ROS pellets were resuspended in 10 mM Tris-HCl (pH 7.4) containing 100 mM NaCl and 1 mM EDTA, and stored at – 20 °C.

#### Metabolic labeling and GC/MS analysis

The strategies and methods we use for quantifying metabolites in retinas have been described in detail^31,32^. Individual retinas isolated mid-morning from ambient light-adapted mice were incubated with 5 mM U-^13^C glucose in an incubator at 5% CO_2_/21% O_2_/balance N_2_, at 37 °C. After 5 min, each retina was quenched in ice-cold saline and homogenized in methanol/chloroform/water. Metabolites were extracted, dried, derivatized, and analyzed by GC–MS as described^31,32^. Data were analyzed using Chemstation software (Agilent Technologies). The measured distributions of mass isotopomers were corrected for natural abundance of ^13^C using IsoCor software^33^.

#### Electroretinography

Flash ERGs were recorded with the Diagnosys Espion E2 ERG system (Diagnosys, LLC, Lowell, MA). Mice were maintained in total darkness overnight and prepared for ERG recording under dim red light. They were anesthetized with ketamine (80–100 mg/kg body weight) and xylazine (5 mg/kg body weight) intramuscularly. One drop of 10% (v/v) phenylephrine was applied to the cornea to dilate the pupil and one drop of 0.5% (v/v) proparacaine HCl was applied for local anesthesia. Mice were kept on a heating pad at 37 °C during recordings. A gold electrode was placed on the cornea, a reference electrode was positioned in the mouth, a ground electrode was placed on the foot, and the mice were placed inside a Ganzfeld illuminating sphere. Responses were differentially amplified, averaged, and stored. For the assessment of rod photoreceptor function (scotopic ERG), we used − 3.4, − 2.4, − 1.4, − 0.4, 0.6, 1.6, 2.6 log cd.s/m^2^; to measure cone function (photopic ERG) we used 3.3 log cd.s/m^2^ flash intensities. The amplitude of the a-wave was measured from the pre-stimulus baseline to the a-wave trough. The amplitude of the b-wave was measured from the trough of the a-wave to the peak of the b-wave. For the evaluation of cone function (photopic ERG), a strobe flash stimulus was presented to dilated, light-adapted mice. The amplitude of the cone b-wave was measured from the trough of the a-wave to the peak of the b-wave.

#### Optical coherence tomography

OCT was used to acquire, process, display, and save in-depth resolved images of the ocular tissue. This approach allows for real-time non-invasive imaging of internal tissue microstructure, such as retina, while the cornea, sclera, and conjunctiva are also viewable with just a change in the focal position. OCT analysis was performed on the Bioptigen SDOIS, which is ideally suited to image the retina in small animals, such as rodents. Mice were anesthetized with an intramuscular injection of a mixture of ketamine (80–100 mg/kg) and xylazine (5 mg/kg), and their pupils were dilated with 1% tropicamide. The mice were placed in the animal mount and the eyes were well hydrated with Genteal eye drops. The extra-ocular lens was positioned and the image was viewed on the monitor in real time. After the OCT analysis, the mice were monitored until they fully recovered from the anesthesia.

#### Pde6β promoter activity

To determine whether PKM2 regulates expression of PDE6β, we PCR-amplified the PDE6β proximal 5′-region from human genomic DNA with sense:5'-GATATCAGCAGAAAGCGTCATGCTG-3' and antisense: 5'-AGATCTGTGGCTGCCTGTCCCTGG-3' primers, and cloned into a sequencing vector. Upon sequencing, the genomic fragment was excised with *EcoRV* and *BglII* enzymes, and cloned into *SmaI* and *BglII* sites of pGL2-luciferase vector containing SV40 promoter and pGL3-luciferase vector containing no promoter. Either empty or pde6β-SV40 promoter or pde6β promoter vectors were transfected into HEK-293T cells by calcium phosphate method with a vector carrying β-galactosidase and with or without Flag-tagged PKM2 constructs. Forty eight hours later, cells were harvested and lysed with buffer obtained from Promega (Madison, WI). Lysates were measured for luciferase and β-galactosidase activities using Promega Kits, and the luciferase activity were normalized to β-galactosidase activity.

#### Preparation of tissue for paraffin sectioning using Prefer as a fixative

Prefer solution (Anatech Ltd, Battle Creek, MI) was used to fix the mouse eyes for 15 min at room temperature, followed by 70% ethanol overnight. The tissue was paraffin embedded, and 5 μm-thick sections were cut and mounted onto slides. These sections were subjected to immunohistochemistry. A Nikon Eclipse E800 microscope equipped with a digital camera was used to examine the antibody-labeled complexes. Metamorph (Universal Imaging, West Chester, PA) image analysis software was used to capture images under identical microscope and camera settings.

#### Immunoblot analysis

Mouse retinas were homogenized in a lysis buffer containing 1% Triton X-100, 137 mM NaCl, 20 mM Tris-HCl (pH 8.0), 10% glycerol, 1 mM EGTA, 1 mM MgCl_2_, 1 mM PMSF, 0.2 mM Na_3_VO_4_, 10 µg/ml leupeptin, and 1 µg/ml aprotinin^34^. Insoluble material was removed by centrifugation at 17,000 × *g* for 20 min at 4 °C. The protein concentrations of the solubilized proteins were determined with the bicinchoninic acid reagent following the manufacturer’s instructions (Pierce Biotechnology, Rockford, IL). Five micrograms of retinal proteins were run on 10% SDS-polyacrylamide gel electrophoresis, followed by protein blotting onto nitrocellulose membranes. After blocking the membranes with 5% non-fat dry milk power (Bio-Rad) or 5% bovine serum albumin (Sigma) for 45–60 min at room temperature, blots were incubated with anti-opsin (1 : 10,000), anti-pPKM2-Y105 (1 : 1,000), anti-PKM2 (1 : 1,000), anti-PKM1 (1:1000), anti-rod arrestin (1 : 1,000), anti-M-opsin (1 : 1,000), anti-cone arrestin (1 : 1,000), anti-Trα (1 : 1,000), anti-PDE6β (1 : 1,000), anti-PDEγ (1 : 1,000), anti-CNGA1 (1 : 1,000), anti-RGS9-1 (1 : 1,000), anti-Gβ5L/S (1 : 1,000), anti-R9AP (1 : 1,000), anti-actin (1 : 1,000), and anti-Glut1 (1 : 1,000) overnight at 4 °C. The blots were then washed and incubated with horseradish peroxidase-coupled anti-mouse, anti-rabbit, or anti-goat secondary antibodies for 60 min at room temperature. After washing, blots were developed with enhanced SuperSignal West Dura Extended Duration Substrate (Thermo Fisher Scientific, Waltham, MA) and visualized using a Kodak Imager with chemiluminescence capability. Densitometric analysis of immunoblots was performed in the linear range of detection. Absolute values were then normalized to arrestin, and statistical analysis was carried out.

#### Phospholipid molecular species analysis of photoreceptor OSs

The methods have been described previously^35,36^. Briefly, ROSs were diluted 1 : 40 with 2-propanol/methanol/chloroform (4 : 2 : 1 v/v/v) containing 20 mM ammonium formate and 1.00 µM PC 14 : 0/14 : 0, 1.00 µM PE 14 : 0/14 : 0, and 0.33 µM PS 14 : 0/14 : 0 as internal standards. Samples were introduced into a triple quadrupole mass spectrometer (TSQ Ultra, Thermo Scientific) using a chip-based nano-ESI source (Advion NanoMate) operating in infusion mode. PC lipids were measured using precursor ion scanning of m/z 184. PE lipids were measured using neutral loss scanning of m/z 141. PS lipids were measured using neutral loss scanning of m/z 185. Quantification of lipid molecular species was performed using the Lipid Mass Spectrum Analysis software’s peak model fit algorithm. Data are represented as relative percent of each measured species within each class (i.e., PC, PE, PS) ± SD.

#### Phospholipid and triglyceride synthesis

PKM2 wild-type and rod-cre PKM2-KO mouse retinas were incubated in Ringer’s solution in the presence of 100 µCi [2-^3^H] Glycerol (DuPont/NEN) for 45 min. Total lipids were extracted^37^ and subjected to thin layer chromatography^38^. Phospholipid and triglyceride spots were scraped from the plate and individually counted using a Beckman LS6000 IC scintillation counter.

#### Statistical analysis

One-way analysis of variance and post-hoc statistical analysis using Bonferroni’s pairwise comparisons were used to determine statistical significance (*p *< 0.05).

## Electronic supplementary material

Supplementary Figure legends

supplementary Figures
