# Supplementary material for: Pyruvate kinase M2 regulates photoreceptor structure, function, and viability
Source: Cell Death Dis. 2018 Feb 14;9(2):240. doi: 10.1038/s41419-018-0296-4 (PMC5833680; doi:10.1038/s41419-018-0296-4)
Supplement: Supplementary file 1 — Supplementary Figure legends [file 41419_2018_296_MOESM1_ESM.docx]

**Supplementary Figures**

**Figure S1. Function of rod PKM2-KO mouse retina.** Scotopic a-wave, scotopic b-wave, and photopic b-wave electroretinographic analysis of wild-type and PKM2-KO mouse retinas at 2 months of age. Scotopic a-and b-wave amplitudes were measured at a flash intensity of 2.6 log cd s/m² whereas photopic b-wave amplitude was measured at a flash intensity of 3.3 log cd s/m². Data are mean + *SEM* (*n=6*).

**Figure S2, Elevated cGMP levels in rod-cre PKM2 KO mouse retinas.** Prefer-fixed retinal sections were subjected to antigen retrieval with 10 mM citrate buffer, PH 6.0. Wild-type (**A**) and rod-cre PKM2-KO (**B**) mouse retinal sections were subjected to immunofluorescence with anti-cGMP (**A, B**) antibody, and nuclei were stained with DAPI. Panel **C** represents the omission of primary antibody. Scale bar = 20 µm.

**Figure S3. Phospholipid analysis of photoreceptor outer segments.** Rod outer segments (ROS) were prepared from wild-type and rod-cre PKM2-KO mice using discontinuous sucrose gradient centrifugation. ROS PC (A), PE (B), and PS (C) lipid molecular species were measured using a triple quadrupole mass spectrometer. Quantification of lipid molecular species was performed using the Lipid Mass Spectrum Analysis (LIMSA) software’s peak model fit algorithm. Data are represented as relative percent of each measured species within each class (i.e., PC, PE, PS). Dare are ± *SD* (*n*=5). **p*<0.05.
