# Supplementary material for: Pyruvate kinase M2 regulates photoreceptor structure, function, and viability
Source: Cell Death Dis. 2018 Feb 14;9(2):240. doi: 10.1038/s41419-018-0296-4 (PMC5833680; doi:10.1038/s41419-018-0296-4)
Supplement: Supplementary file 2 — supplementary Figures [file 41419_2018_296_MOESM2_ESM.pdf]

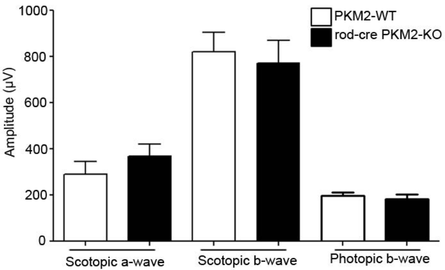
1000
800
600
400
200
0
Amplitude (μV)
PKM2-WT
rod-cre PKM2-KO
Scotopic a-wave
Scotopic b-wave
Photopic b-wave

Figure S1

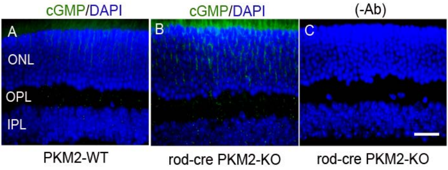
cGMP/DAPI
cGMP/DAPI
(-Ab)
A
ONL
OPL
IPL
B
C
PKM2-WT
rod-cre PKM2-KO
rod-cre PKM2-KO

Figure S2

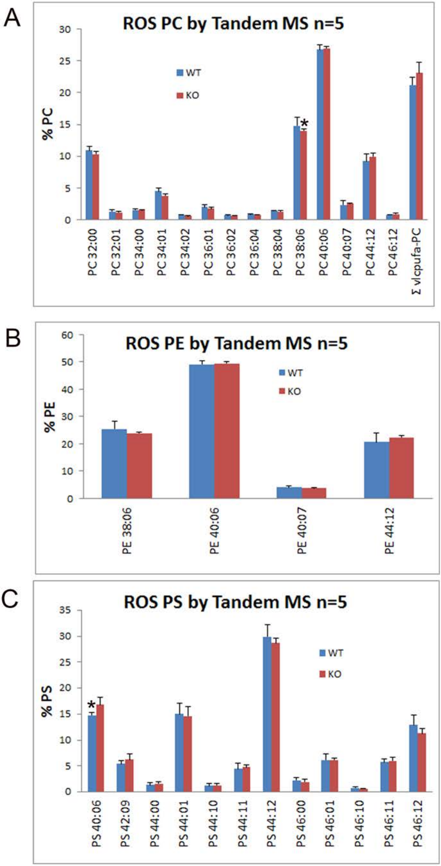
A
ROS PC by Tandem MS n=5
% PC
WT
KO
PC 32:00
PC 32:01
PC 34:00
PC 34:01
PC 34:02
PC 36:01
PC 36:02
PC 36:04
PC 38:04
PC 38:06
PC 40:06
PC 40:07
PC 44:12
PC 46:12
Σ vlcpufa-PC
B
ROS PE by Tandem MS n=5
% PE
WT
KO
PE 38:06
PE 40:06
PE 40:07
PE 44:12
C
ROS PS by Tandem MS n=5
% PS
WT
KO
PS 40:06
PS 42:09
PS 44:00
PS 44:01
PS 44:10
PS 44:11
PS 44:12
PS 46:00
PS 46:01
PS 46:10
PS 46:11
PS 46:12

Figure S3
